# Nasal Mucous Membrane as a Remedy

**Published:** 1897-11

**Authors:** 


### Nasal Mucous Membrane as a Remedy.

Dr. Reviere, of Lyons (*Lyon Medical*, September 19th), reports that he has employed in the treatment of a certain number of nasal affections a fluid extract of the pituitary mucous membrane prepared by Dr. Jacquet in the following manner: The mucous membrane of the middle and lower terbinated bones of the sheep is macerated for twenty-four hours, at a temperature kept at 159° F., in water containing four parts of resorcin in a thousand; the liquid is then filtered and subjected to the same degree of heat for twenty-four hours more. The results of the use of this preparation, says Riviere, are analogous to those produced with other substances that are efficient in cases of perforation of the sæptum, rhinitis sicca, and rebellious syphilitic disease of the nose. In a grave case of ozæna that had relapsed after various sorts of treatment, including the employment of electrolysis, applications of the pituitary extract, after cleansing, were followed by a rapid subsidence of the odor and then by greater benefit in every way than is generally obtained by the use of procedures less innocent or more difficult. —*Ex.*

